# In the Solanaceae, a hierarchy of bHLHs confer distinct target specificity to the anthocyanin regulatory complex

**DOI:** 10.1093/jxb/eru494

**Published:** 2015-01-26

**Authors:** Mirco Montefiori, Cyril Brendolise, Andrew P. Dare, Kui Lin-Wang, Kevin M. Davies, Roger P. Hellens, Andrew C. Allan

**Affiliations:** ^1^The New Zealand Institute for Plant & Food Research Ltd, Private Bag 92 169, Auckland, New Zealand; ^2^The New Zealand Institute for Plant and Food Research Ltd, Private Bag 11 600, Palmerston North, New Zealand; ^3^Biochemistry Department, School of Medical Sciences, University of Otago, Dunedin 9054, New Zealand; ^4^Centre for Tropical Crops and Biocommodities Queensland University of Technology Brisbane, Queensland, Australia; ^5^School of Biological Sciences, University of Auckland, Private Bag 92019, Auckland, New Zealand

**Keywords:** bHLH, MYB, Solanaceae, tobacco, transcriptional control, transcription factors.

## Abstract

The anthocyanin biosynthetic pathway is regulated by a transcription factor complex consisting of an R2R3 MYB, a bHLH, and a WD40. Although R2R3 MYBs belonging to the anthocyanin-activating class have been identified in many plants, and their role well elucidated, the subgroups of bHLH implicated in anthocyanin regulation seem to be more complex. It is not clear whether these potential bHLH partners are biologically interchangeable with redundant functions, or even if heterodimers are involved. In this study, AcMYB110, an R2R3 MYB isolated from kiwifruit (*Actinidia* sp.) showing a strong activation of the anthocyanin pathway in tobacco (*Nicotiana tabacum*) was used to examine the function of interacting endogenous bHLH partners. Constitutive expression of *AcMYB110* in tobacco leaves revealed different roles for two bHLHs, *NtAN1* and *NtJAF13*. A hierarchical mechanism is shown to control the regulation of transcription factors and consequently of the anthocyanin biosynthetic pathway. Here, a model is proposed for the regulation of the anthocyanin pathway in Solanaceous plants in which AN1 is directly involved in the activation of the biosynthetic genes, whereas JAF13 is involved in the regulation of AN1 transcription.

## Summary

Anthocyanin biosynthesis is regulated by a transcription factor complex. Here, it is determined that the potential bHLH partners in this complex function in a hierarchy to control each other and the anthocyanin biosynthesis pathway.

## Introduction

Anthocyanins are the major pigmented group of flavonoid compounds responsible for a range of colours varying from pink to blue, through to red and violet in flowers, fruits, or leaves. The anthocyanin biosynthetic pathway is one branch of flavonoid metabolism (Winkel-Shirley, 2001) and its regulatory system has been extensively studied. The anthocyanin biosynthetic genes are transcriptionally regulated by a MYB–bHLH–WD40 (MBW) complex that contains R2R3 MYB and basic helix-loop-helix (bHLH) transcription factors and a WD40 protein (Feller *et al.*, 2011). The expression patterns of the MBW components thus define the variety of pigmentation patterns of plant tissues (Davies *et al.*, 2012),

The R2R3 MYBs are numerically the largest class of transcription factors in plants. The *C1* MYB (*Zea mays*) was the first transcription factor identified to regulate anthocyanin biosynthesis (Paz-Ares *et al.*, 1987); subsequently a large number of MYBs controlling anthocyanin colour in flowers (Quattrocchio *et al.*, 1999; Schwinn *et al.*, 2006; Albert *et al.*, 2011) and fruit (Kobayashi *et al.*, 2004; Takos *et al.*, 2006; Espley *et al.*, 2007; Lin-Wang *et al.*, 2010) have been identified.

Basic helix-loop-helix proteins are the second largest class of transcription factor. Generally, the first 200 amino acids of the protein are involved in the interaction with the MYB partner, whereas the following 200 amino acids interact with the WD40 protein. The bHLH domains are involved in the formation of homo- or heterodimers with other bHLH proteins, which often is a prerequisite for DNA recognition and contributes to DNA-binding specificity (Feller *et al.*, 2011; Hichri *et al.*, 2011). The bHLH group of transcription factors have been divided into 26 subgroups (Pires and Dolan, 2010), but if ‘atypical’ bHLHs are included this is extended to 32 subgroups (Carretero-Paulet *et al.*, 2010). Flavonoid related bHLHs have been grouped into subgroup IIIf.

The first bHLHs regulating the flavonoid pathway were identified in maize, *Booster1* (B) and *Red1* (R) (Chandler *et al.*, 1989). Subsequently a number of additional bHLH transcription factors able to regulate the flavonoid pathway have been identified: these include, *AN1* and *JAF13* in *Petunia* (*Petunia hybrida)* (Quattrocchio *et al.*, 1998; Spelt *et al.*, 2000), *Delila* and *Mutabilis* in snapdragon (*Antirrhinum majus)* (Martin *et al.*, 1991), *TT8* and *GLABRA3* in *Arabidopsis* (Nesi *et al.*, 2000; Feyissa *et al.*, 2009), *VvMYC1* and *VvMYCA1* in grape (*Vitis vinifera*) (Hichri *et al.*, 2010) and *A* in pea (*Pisum sativum*) (Hellens *et al.*, 2010). Many of these have been shown to regulate different physiological or morphological events including different branches of the flavonoid pathway, vacuole acidification, and epidermal cell fate (Hichri *et al.*, 2011).

The interaction between the MYB and bHLH proteins has often been used to study combinatorial gene regulation in plants. In maize the presence of an amino acid motif in the R3 region of the MYB, identified as (DE)Lx_2_(RK)x_3_Lx_6_Lx_3_R, determines the binding specificity of the MYB to the bHLH partner and is responsible for the different ability of *ZmC1* and a related R2R3 MYB, *ZmP1*, to interact with the bHLH *R*. The ability of the MYB to interact with the bHLH triggers the activation of the biosynthetic gene *ZmBz1*, with *R* playing a key role in the target specificity of the MBW complex (Grotewold *et al.*, 2000; Zimmermann *et al.*, 2004).

In *Petunia*, the MBW complex *AN2–AN1–AN11* (MYB–bHLH–WD40, respectively) regulates anthocyanin biosynthesis in the flower petals (deVetten *et al.*, 1997; Quattrocchio *et al.*, 1999; Spelt *et al.*, 2000). *AN1* and *AN11* are also involved in the control of other physiological events such as acidification of the vacuole when associated in a MBW complex that involves the MYB *Ph4* (Quattrocchio *et al.*, 2006). In *Petunia*, two bHLHs have been identified: *JAF13* and *AN1*. Both bHLHs have been shown to regulate anthocyanin biosynthesis in *Petunia* flowers (Quattrocchio *et al.*, 1998; Spelt *et al.*, 2002) and they have often been used interchangeably in functional assays. However, their role in anthocyanin regulation has not been fully resolved, and they have been shown to have functional differences. In mutant lines, such as the *an1* ^*–*^ mutant (Spelt *et al.*, 2000) and in over-expression lines, *JAF13* is not able to complement the lack of *AN1*.

It seems likely that the MBW complex includes different classes of MYBs and bHLHs with specific functions in regulating the transcription of the flavonoid pathway. Alternatively, different bHLHs may be involved as heterodimers in the MBW complex to regulate the anthocyanin pathway. This specificity of functions amongst similar transcription factors, or involvement of multiple bHLH classes, would allow for more subtle regulation of the transcriptional mechanism through different combinations of transcription factors within the MBW complex. It is therefore necessary to understand how apparently similar MYB or bHLH transcription factors within the MBW complex can have differing regulatory activities.

Here it is shown that *AcMYB110*, an R2R3 MYB from kiwifruit (Fraser *et al.*, 2013), elicits strong induction of anthocyanin when transiently expressed in the model plant tobacco (*Nicotiana tabacum).* This response enables us to further define the specific roles of the bHLH proteins NtAN1 and NtJAF13 and to present a model that explains the hierarchical interactions of the MYB and bHLH partners within the MBW complex.

## Materials and methods

### Phylogenetic analysis

Protein alignment used ClustalW within the software package Geneious 6.1.7 (Biomatters Ltd., Auckland, New Zealand), with manual refinement. A phylogenetic tree was formed from the alignment within the Geneious package using PhyML v2.2 and the maximum likelihood method (Guindon and Gascuel, 2003), with the default settings (NNIs tree topology search, BioNJ initial tree, LG model for amino acids substitutions, and 1000 bootstrap replicates).

### Transient assay

Transient assays were performed as described previously (Hellens *et al.*, 2005) using tobacco (*Nicotiana tabacum*) ‘Samsun’ and *Nicotiana benthamiana* plants grown in a glasshouse, using natural light with daylight extended to 16h. Transient dual-luciferase and colour assay were executed infiltrating approximately 300 µl of *Agrobacterium tumefaciens* GV3101 (MP90) mixture into young leaves. *Agrobacterium* were cultured on Lennox agar plates supplemented with selection antibiotics and incubated at 28 °C. The freshly grown bacteria were re-suspended in infiltration media and incubated at room temperature for at least 2h (Hellens *et al.*, 2005) before infiltration.

To generate overexpression vectors the open reading frames of *AcMYB110* and *NtAN1* were amplified using specific primers (Supplementary Table S1) and cloned into pENTR-TOPO and subsequently a Gateway reaction was performed with destination expression vector pHEX2 (http://www.lifetechnologies.com). *PhAN1* expression vector, as described in Albert *et al.* (2011), was provided by Nick Albert (Plant and Food Research, Palmerston North, NZ).

To generate the RNA interference constructs, small fragments were amplified of *NtAN1* and *NtJAF13* (respectively 383bp and 197bp long) with specific primers (Supplementary Table S1) and the cloned products were put into pENTR-TOPO, followed by a Gateway reaction with RNAi destination vector pTKO2 (http://www.lifetechnologies.com).

For the colour assay, a mixture of *Agrobacterium* transformed with constructs constitutively expressing transcription factors fused to the 35S promoter in pHEX2 and silencing the endogenous bHLHs *NtAN1* and *NtJAF13* by RNAi using a pTKO2 vector was used. Final colour development was assayed at four days and seven days after infiltration.

Tobacco *AN1*, *CHS*, and *DFR* promoter sequences were obtained by a genome walking strategy; approximately 1kb of target promoter was amplified using specific primers (Supplementary Table S1) and inserted into pGreen 0800-LUC. Promoter activation assays were performed in tobacco leaves. *Agrobacterium* containing the promoter–LUC fusions were mixed with *Agrobacterium* containing the transcription factor to be tested (ratio 1:3) and used to infiltrate tobacco leaves (Hellens *et al.*, 2005). The resulting luminescence was measured four days after infiltration.

### Pigment analysis

Five independent replicates were collected for each infiltration. Leaf samples were freeze-dried and ground. Anthocyanins were extracted in acidified MeOH (0.1% HCl) and centrifuged at 3000g. Aliquots were dried down and re-suspended in 20% v/v aqueous MeOH (0.2ml). The re-dissolved extracts were analysed by reversed phase HPLC using a method previously described (Andre *et al.*, 2012). Anthocyanin peaks were identified by comparison of retention times with authentic standards and absorption at 520nm. Total anthocyanin content was calculated using standard solutions of cyanidin-3 galactoside (Extrasynthese, Genay, France) and expressed as cyanidin galactoside equivalents ng mg^–1^ of fresh weight.

### Real time qPCR analysis

Gene expression analysis was performed on infiltrated leaves, 4 d after infiltration. RNA was isolated using TRIzol® (http://www.lifetechnologies.com) and DNAse-treated with DNA-free:Ambion (http://www.ambion.com/). Reverse transcription was performed using oligo(dT) according to the manufacturer’s protocol (Transcriptor: Roche Diagnostics, http://www.roche.com/). Quantitative real-time PCR was carried out using the LightCycler 480 System, using 480 SYBR® Green I Master (Roche Diagnostics). Reactions were performed in quadruplicate and a non-template control was included in each run. The data were analysed using the LightCycler® 480 software 1.5 normalised to tobacco actin (GQ281246) and calibrated to the control (empty vector) infiltration (primer sequences in Supplementary Table S1).

### Yeast one-hybrid and yeast two-hybrid assays

Yeast one-hybrid experiments were conducted using the Matchmaker Gold Y1H library Screening System (Clontech). 490bp and 562bp of the *NtAN1* and *NtDFR* promoters, respectively, were cloned in the pAbAi vector and transformed into Y1HGold strain to generate the bait reporter strains. *NtAN1* and *NtJAF13* full-length coding sequence were cloned into pDEST22 (Life Technologies) and transfected into yeast as a fusion to the GAL4 activating domain (AD). MYB110 was cloned using Gateway recombination technology (Life Technologies) in pGW6, a Gateway-adapted version of pTFT1 vector (Egea-Cortines *et al.*, 1999) which allows expression of an untagged protein in yeast. The different pairwise combinations of MYB and bHLHs and corresponding empty vector controls were co-transformed into the Y1HGold strain containing the promoter construct. Growth on minimal synthetic defined media (SD) lacking adenine, uracil, and tryptophan and supplemented with 50ng ml^–1^ aureobasidin A was scored after 5 d at 30 °C.

For the yeast two-hybrid assays, full length sequences of the candidate genes were inserted in pDEST22 and pDEST32 vectors from the ProQuest™ Two-Hybrid System (Life Technologies) and transfected in the PJ69-4A and PJ69-4alpha yeast strains (James *et al.*, 1996). Pairwise combinations of MYBs and bHLHs, expressed as fusion proteins to the GAL4 activating (AD) and binding (BD) domains respectively, were obtained by mating and screened for growth after up to 3 d at 30 °C on SD media lacking tryptophan (T), leucine (L) and histidine (H) and supplemented by increasing amount of 3-amino-1,2,4-triazole (3AT) (10, 25, 50, 75, and 100mM) to increase the stringency of the histidine selection (Supplementary Fig. S1).

### Statistical analysis

Analysis of variance (ANOVA) using the Genstat 14th edition, Tukey test (*P*<0.05) was used to separate the treatment means.

## Results

### Phylogenetic analysis of anthocyanin-related bHLHs

**Fig. 1.**
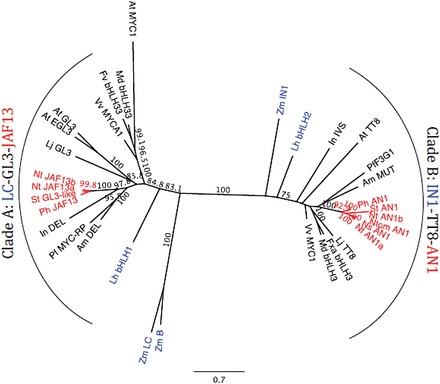
Phylogenetic tree of bHLH transcription factors involved in the regulation of the flavonoid pathway from a range of species. Amino acid sequences were aligned using ClustalW and a maximum likelihood tree formed from the alignment. Numbers indicate bootstrap values for 1000 replicates, with only values above 70 shown. Accession details are: *Antirrhinum majus* DELILA (AAA32663), MUTABILIS (Schwinn *et al.*, 2006); *Arabidopsis thaliana* EGL3 (Q9CAD0), GL3 (NP_680372), MYC1 (Q8W2F1), TT8/bHLH42 (Q9FT81); *Fragaria vesca* bHLH33 (XP_004308377); *Fragaria × ananassa* bHLH3 (AFL02463); *Ipomoea nil* DEL (BAE94393), IVS (ivory seeds, BAE94394); Lilium (hybrid division I) bHLH1 (BAE20057), bHLH2 (BAE20058); *Lotus japonicus* GL3 (AB492284), TT8 (AB490778); *Malus domestica* bHLH3 (ADL36597), bHLH33 (ABB84474); *Nicotiana sylvestris* AN1 (HQ589210); *Nicotiana tabacum AN1a* (HQ589208), *AN1b* (HQ589209), *JAF13a* (KF305768), *JAF13b* (KF298397); *Nicotiana tomentosiformis* AN1 (HQ589211); *Petunia x hybrida* AN1 (AAG25928), JAF13 (AAC39455); *Perilla frutescens* F3G1 (AB103172), MYC-RP (AB024050); *Solanum tuberosum* AN1 (JX848660), GL3-like (NM_001288203); *Vitis vinifera* MYC1 (ACC68685), MYCA1 (ABM92332); *Zea mays* B-PERU (CAA40544), IN1 (AAB03841), LC (P13526). Sequences from Solanaceous species are shown in red and those from monocotyledonous species in blue.

Phylogenetic analysis, performed on the deduced amino acid sequences of more than 30 flavonoid-related bHLHs belonging to the subgroup IIIf, clearly separates them in two clades (Fig. 1): clade A and clade B, as described in Davies *et al.* (2012). Clade A included JAF13 (*Petunia*), *R* (maize), Delila (snapdragon), and GL3/EGL3 (*Arabidopsis*), whereas clade B contained AN1 (*Petunia*), Intensifier (maize), Mutabilis (snapdragon), and TT8 (*Arabidopsis*). Members of the same subgroups are often involved in the same biological process, although it is not always clear if their functions overlap. For example; GL3, EGL3, and TT8 are partially redundant in the control of anthocyanin biosynthesis in *Arabidopsis*. However, each of these have also more specific functions such as PA biosynthesis (TT8), trichome formation (GL3/EGL3), and production of seed coat mucilage (TT8/EGL3) (Feller *et al.*, 2011). Moreover, in *Petunia* the lack of AN1 (clade B) cannot be complemented by JAF13 (clade A), despite both being able to regulate anthocyanin biosynthesis (Spelt *et al.*, 2000). On the other hand, in snapdragon Delila and Mutabilis, respectively from clade A and clade B, seem to be functionally redundant and able to complement for the loss of the other factor (Schwinn *et al.*, 2006).

### Ectopic expression of AcMYB110 reveals different abilities to promote red pigmentation depending on the availability of endogenous bHLHs

**Fig. 2.**
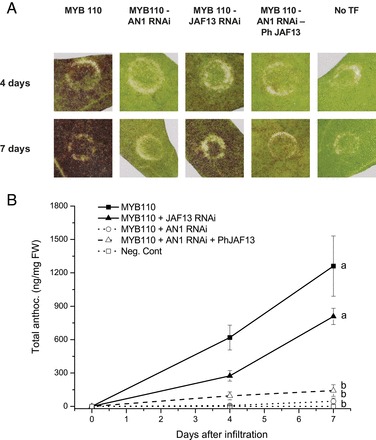
(A) Red colour development in *Nicotiana tabacum* leaves 7 days after ectopic expression of *AcMYB110* in combination with bHLH RNAi constructs targeting tobacco *NtAN1* and *NtJAF13*, and constructs constitutively expressing *Petunia hybrida PhJAF13*. (B) Anthocyanin content in infiltrated patches 4 and 7 days after infiltration. Anthocyanin content is expressed as cyanidin galactoside equivalents ng mg^–1^ of fresh weight. Error bars are SE for five replicates.

When anthocyanin-related MYBs are ectopically expressed transiently in leaves of tobacco (*Nicotiana tabacum*) an anthocyanic patch can be generated (Espley *et al.*, 2007). In the current study, kiwifruit *AcMYB110* (Fraser *et al.*, 2013) was used because of its strong ability to promote anthocyanin biosynthesis in tobacco leaves without the need to provide ectopic expression of a bHLH partner. This ability of *AcMYB110* to activate the anthocyanin pathway of tobacco when transient expressed on its own is different from many other anthocyanin-related R2R3 MYBs (Lin-Wang *et al.*, 2010). Four days after infiltration red pigmentation was evident, and this developed into a dark red patch after 7 days (Fig. 2).

This ability of *AcMYB110* to induce anthocyanin biosynthesis, without co-infiltration of a bHLH, could be due to an efficient interaction with the endogenous tobacco bHLH and WD40 proteins. To investigate these interactions, RNA interference (*RNAi*) was used to inhibit the activity of each of the endogenous tobacco bHLHs known to be involved in anthocyanin biosynthesis: *NtAN1a* (HQ589208), *NtAN1b* (HQ589209), and *NtJAF13b* (KF298397). When *NtAN1* was silenced (the same RNAi construct was able to target both *NtAN1a* and *NtAN1b*) no red colour occurred, despite constitutive expression of *AcMYB110* (Fig. 2A, B). In contrast constitutive expression of *AcMYB110* and *NtJAF13* RNAi still resulted in a red patch, although knock-down of *NtJAF13b* had the effect of delaying the development of the pigmentation (Fig. 2A, B). Four days after infiltration the anthocyanic patches where *AcMYB110* and *NtJAF13 RNAi* were co-infiltrated were less intense compared with the darker pigmentation of the leaves infiltrated with *AcMYB110* only, and even after 7 days they did not reach the same anthocyanin content of *AcMYB110* only (Fig. 2A, B).

These results demonstrated that, in the tobacco system, AcMYB110 requires NtAN1 to activate anthocyanin biosynthesis, whereas the lack of NtJAF13b results in a slower development of pigmentation (Fig. 2A, B). A related phenotype is seen in colour development of flowers of stable RNAi tobacco lines targeting endogenous *NtAN1*(*a* and *b*) and *NtJAF13b*. *NtAN1* RNAi lines developed white flowers, whereas the *NtJAF13* RNAi lines developed less intense pink flowers compared with the wild type (Supplementary Fig. S2). To further characterize the functional interaction of the two bHLHs, the down-regulated *NtAN1* (*NtAN1 RNAi*) was complemented by constitutively expressing the *Petunia hybrida JAF13* (*PhJAF13*; AF020545). Using the *Petunia PhJAF13* to complement the reduction in expression of the tobacco bHLH allows the monitoring by qPCR of expression of the endogenous *NtbHLH*s, as there is sufficient sequence difference between the genes from the two species. Constitutive expression of *PhJAF13* was not able to complement the lack of tobacco *NtAN1* (*RNAi*) and did not restore the ability of *AcMYB110* to drive red pigmentation (Fig. 2). This demonstrates distinct functional properties for the two bHLHs and suggests differing roles in the anthocyanin regulatory system.

### Ectopic expression of AcMYB110 up-regulates tobacco NtAN1 and the biosynthetic genes of the anthocyanin pathway in distinct ways

**Fig. 3.**
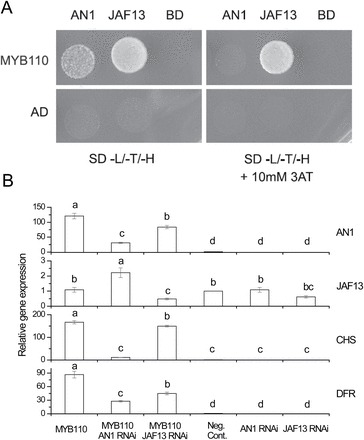
Interaction of MYB110 with different bHLH partners. (A) Analysis of MYB110, AN1, and JAF13 protein–protein interactions by yeast two-hybrid. Pairwise combinations (as indicated) were tested for their ability to activate the HIS3 reporter in the presence of 0 or 10mM 3AT. SD, minimal media; AD, activating domain only; BD, binding domain only; L, leucine; T, tryptophan; H, histidine. (B) Gene expression analysis of endogenous tobacco bHLHs: *NtAN1*, *NtJAF13*, and the anthocyanin biosynthetic genes *chalcone synthase* (*CHS*) and *dihydroflavonol 4-reductase* (*DFR*) 4 days after transient tobacco transformation constitutively expressing *MYB110* with *NtAN1* and *NtJAF13* RNAi constructs. Error bars are SEM for four replicate reactions. Relative quantification is normalized to *NtActin* and calibrated to the ‘no MYB’ infiltration control

AcMYB110 is able to form a complex with either NtAN1 or NtJAF13, as confirmed by yeast two-hybrid assay (Fig. 3A). NtJAF13 and NtAN1 may target the MBW complex to different promoters within this pathway. The gene expression of chalcone synthase (*NtCHS*; AB213651) and dihydroflavonol 4-reductase (*NtDFR*; EF421429) were therefore examined, as representative of early and late stages of anthocyanin biosynthesis; as well as the expression of the endogenous *NtJAF13b* and *NtAN1* genes. Ectopic expression of *AcMYB110* in the tobacco leaf up-regulated the transcript level of the endogenous *NtAN1* and the biosynthetic genes *NtCHS* and *NtDFR* (Fig. 3B). This is consistent with the development of a red pigmentation of the infiltrated leaf patches. In contrast, *AcMYB110* did not have a significant effect on the expression of tobacco *NtJAF13.*

As expected, co-infiltration of the *NtAN1 RNAi* with *AcMYB110* strongly reduced the transcript level of the endogenous *NtAN1* and consequently the expression of the biosynthetic genes of the pathway and the colour of the leaf patch. On the contrary, *NtJAF13* expression was slightly elevated when RNAi was directed at *NtAN1*. *NtJAF13* RNAi did not completely silence the endogenous *NtJAF13* transcript, but reduced its expression by approximately half. The reduced expression of *NtJAF13* and ectopic expression of *AcMYB110* resulted in a reduced up-regulation of *NtAN1* and consequently of *NtCHS* and *NtDFR* (Fig. 3B).

### AcMYB110 activates the promoters of *NtAN1* and the biosynthetic genes *NtCHS* and *NtDFR*

**Fig. 4.**
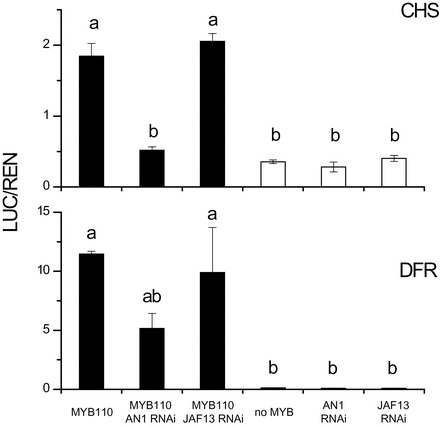
Promoter activation of the biosynthetic gene of the pathway *NtCHS* and *NtDFR* on a dual luciferase transient assay in *Nicotiana tabacum*. Tobacco leaves were infiltrated with *35S*:*MYB110* in different combination with RNAi constructs targeting the endogenous bHLHs *AN1* and *JAF13*. The dual luciferase assay shows promoter activity expressed as a ratio of target promoter luciferase (LUC) to 35S Renilla (REN); a greater activity equates to an increase in LUC relative to REN. Error bars are the SEM for four replicate reactions.

**Fig. 5.**
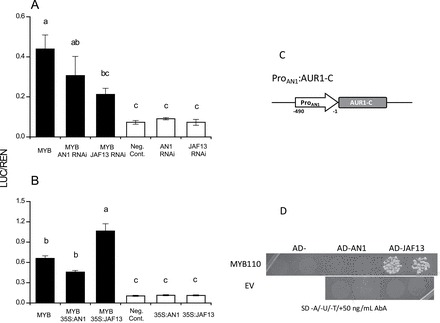
MYB110 interacts with the promoter of *NtAN1.* (A) *NtAN1* promoter activation using a dual luciferase transient assay in tobacco leaves infiltrated with *35S*:*MYB110* in combination with RNAi constructs targeting the endogenous bHLHs *AN1* and *JAF13*. Error bars are the SEM for four replicate reactions. (B) *NtAN1* promoter activation using a dual luciferase transient assay in tobacco leaves infiltrated with *35S*:*MYB110* in combination with *35S:NtAN1* and *35S:JAF13*. Error bars are the SEM for four replicate reactions. (C) Diagram of the yeast one-hybrid reporter construct containing 490bp of the *NtAN1* promoter region. (D) Y1H analysis of MYB110, NtJAF13, and NtAN1 interactions with the *AN1* promoter. Results obtained with two independent colonies for each combination are presented. SD, minimal media; A, adenine; U, uracil; T, tryptophan; AbA, Aureobasidin A; EV, empty vector.

Using a transient dual luciferase assay (Hellens *et al.*, 2005), the ability of AcMYB110 to regulate the tobacco *NtCHS, NtDFR,* and *NtAN1* promoters was tested. Different combinations of *AcMYB110* and RNAi knock-down constructs directed at the endogenous bHLHs were co-infiltrated in tobacco leaves together with the pGREEN-LUC constructs, which contained the target promoters (tobacco *CHS*, *DFR* or *AN1*) driving the expression of the luciferase reporter gene (LUC). The results are presented relative to the level of the Renilla (REN) reporter gene, which is under control of a 35S promoter. As expected, AcMYB110 was able to activate the promoters of the biosynthetic genes, *NtCHS* and *NtDFR* (Fig. 4). However, when *NtAN1* was down-regulated there was a significant drop in the activation of these promoters. Co-infiltration of *NtJAF13* RNAi did not significantly affect *AcMYB110* activation of the *NtCHS* and *NtDFR* promoters. Moreover*, AcMYB110* was able to activate the *NtAN1* promoter (Fig. 5A), and addition of the *NtAN1* RNAi construct had no effect on this activation. In contrast, activation of the *NtAN1* promoter was reduced in the presence of the *NtJAF13* RNAi construct (Fig. 5A). Ectopic expression of *PhJAF13* enhanced activation of the *NtAN1* promoter, whereas over-expression of *NtAN1* did not improve the activation of the *NtAN1* promoter (Fig. 5B).

Direct binding to the *NtAN1* promoter by AcMYB110 was assayed by yeast one-hybrid (Fig. 5C). AcMYB110 was shown to bind the *NtAN1* promoter only in presence of the NtJAF13, whereas no binding was demonstrated in the presence of NtAN1 (Fig. 5D). This result confirms the transient luciferase assay results where NtJAF13 seems to be involved in the regulation of *NtAN1*.

### Comparative *in silico* analysis of the promoter regions of AN1, CHS, and DFR

**Fig. 6.**
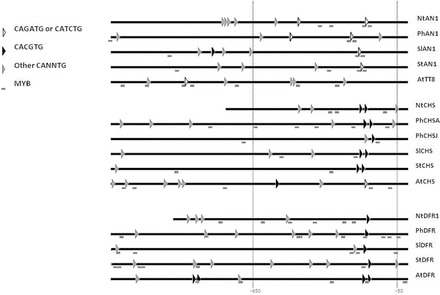
Schematic representation of bHLH and MYB motifs in the promoter regions of the anthocyanin biosynthetic genes (*CHS* and *DFR*) and *AN1*, or *AN1-like*, from *Arabidopsis* and four different Solanaceous species: tobacco, *Petunia*, tomato, and potato. Grey boxes indicate MYB binding sites corresponding to MYBPLANT (MACCWAMC), MYBPZM (CCWACC), MYBST1 (GGATA), and MYBCORE (CNGTTR) consensus motifs from PLACE database. bHLH binding sites corresponding to Ebox (CANNTG) motif are indicated by triangles. CAGATG or CACTG putative bHLH motifs (white triangles) were found specifically in the *AN1* promoters of the four Solanaceae. Conversely, the CACGTG bHLH motifs (black triangles) were only found in the promoter region of the biosynthetic genes of the four Solanaceae and *Arabidopsis*.

The promoter sequences of *N. tabacum NtAN1*, *NtCHS*, and *NtDFR* were analysed using the PLACE database SignalScan program (Higo *et al.*, 1999) and several potential MYB (Dare *et al.*, 2008) and bHLH binding sites were identified (Fig. 6 and Supplementary Fig. S3). Nine different variants of the canonical CANNTG bHLH binding motif were identified. When focusing on the 650bp region upstream of the ATG, it was found that the CACGTG variant was only present in the promoters of the two biosynthetic genes whereas two other motifs, CATCTG and its reverse complement CAGATG, were only found in the *NtAN1* promoter. The promoter analysis of *AN1*, *CHS*, and *DFR* was extended to three other species of the Solanaceae family (*Petunia*, potato, tomato) as well as to *Arabidopsis*. The results confirmed that the CACGTG motif was specific to the *CHS* and *DFR* biosynthetic genes and that either, or both, CATCTG and CAGATG motifs were present in the *AN1-like* promoters in the Solanaceae. However, this did not occur in the *Arabidopsis* promoters as the CATCTG motif was present in the *AtCHS* promoter and none of the two *AN1*-specific motifs were present in the *TT8* promoter. Although the *AN1-like* promoters of the four Solanaceae share a low pairwise identity (48–55% with one another and 81% between tomato and potato AN1), the common CACGTG motif sits in a highly conserved stretch of 27bp which also contains conserved putative MYB binding sites. This suggests that this region is under high selective pressure and so may have an important regulatory function.

## Discussion

The transcriptional regulation of anthocyanin biosynthesis has been well described in plants and involves an MBW complex consisting of an R2R3 MYB, a bHLH, and a WD40 protein. MYBs and bHLHs that regulate the anthocyanin biosynthetic pathway have been extensively described in several plants of the Solanaceae family and are often found as small multi-gene families e.g. *AN1, JAF13, AN2, DPL*, and *PHZ* in *Petunia* (Quattrocchio *et al.*, 1998; Quattrocchio *et al.*, 1999; Spelt *et al.*, 2000; Albert *et al.*, 2011).

Although the different members of the bHLH and R2R3 MYB multi-gene families have often been thought of as having very similar roles, there is increasing evidence of specialization of function. Not only is there considerable amino acid sequence variability between the flavonoid-related MYB genes from the Poaceae (such as *ZmC1*) and those of dicots, but different gene family members within a species can vary in function, as seen from markedly different transgenic phenotypes resulting from over-expression of *PhAN2*, *PhDPL*, or *PhPHZ* in *Petunia* (Quattrocchio *et al.*, 1999; Albert *et al.*, 2011). Less information is available for the subfamily IIIf bHLHs belonging to the two different clades; one that includes sequences such as AN1 (*Petunia*), TT8 (*Arabidopsis*), and Mutabilis (snapdragon), and a second that contains sequences such as JAF13 (*Petunia*), Delila (snapdragon), and GL3 from *Arabidopsis* (Fig. 1).

Here, the roles of the two anthocyanin-related bHLH clades have been investigated using kiwifruit (*Actinidia) AcMYB110*, previously shown to activate anthocyanin biosynthesis (Fraser *et al.*, 2013), and which is able to promote a red anthocyanic patch when expressed transiently without co-expression of an exogenous bHLH. Because of this strong activating ability, *AcMYB110* proved to be a useful tool to study possible functional differences between the two flavonoid-related bHLH clades in tobacco.

### The MYB–AN1 complex directly activates anthocyanin biosynthetic genes

Ectopic expression of the MYB *NtAN2* in tobacco promotes anthocyanin biosynthesis and results in red pigmentation in both vegetative and floral tissues (Pattanaik *et al.*, 2010). This is achieved by up-regulating the expression of the biosynthetic genes and the *NtAN1* bHLH (Bai *et al.*, 2011). Here, similar results were obtained when *AcMYB110* was transiently expressed in tobacco leaves: *NtAN1* was up-regulated, as were the biosynthetic genes of the pathway, resulting in dark red patches only a few days after infiltration (Fig. 2). However, constitutive expression of *AcMYB110* did not affect the expression level of *NtJAF13*. Co-infiltration of *AcMYB110* with the *NtAN1* RNAi construct prevented activation of *NtCHS* and *NtDFR* and therefore red pigmentation (Figs 2, 3B, and 4), and the constitutive expression of *PhJAF13* was not able to complement the lack of *NtAN1* (Fig. 2). This is consistent with previous results where *AN1* was shown to be necessary to activate the anthocyanin pathway: in *Petunia*, a white petal *an1* mutant is due to the lack of *PhAN1* and over-expressing *PhJAF13* was not sufficient to restore anthocyanin biosynthesis (Spelt *et al.*, 2000). A similar situation is found in pea, where loss of function of an *AN1*-like bHLH, corresponding to Mendel’s *A* gene, results in white flowers (Hellens *et al.*, 2010). However, less specificity is seen in other species than that of *Petunia* or tobacco, where phenotypic redundancy between the bHLH family members is seen. For example, in snapdragon the bHLH *Delila* complements for the loss of another anthocyanin-related bHLH, *Mutabilis* (Schwinn *et al.*, 2006). In contrast, co-infiltration of *AcMYB110* with *NtJAF13* RNAi resulted in a reduced level of *NtJAF13* which did not seem to affect the ability of *AcMYB110* to promote anthocyanin accumulation (Figs 2 and 3B).

### The MYB–JAF13 complex has an indirect effect on anthocyanin biosynthesis through regulation of AN1 expression

Gene expression data (Fig. 3B) of the infiltrated leaves supported the role of NtJAF13 as an indirect regulator of the pathway by aiding the activation of *NtAN1* expression by the MYB. Previous work had suggested that anthocyanin-related MYBs can regulate *AN1* expression. In particular, MYB *AN2* (or MYB *AN4*) and *MYB75*-*PAP1* were shown to enhance the expression of *PhAN1* and *TT8* in *Petunia* and *Arabidopsis*, respectively (Spelt *et al.*, 2000; Tohge *et al.*, 2005). Moreover, there seems to be some level of functional redundancy amongst the flavonoid-related MYBs in regulating *AN1* expression, given that *PhAN1* is still expressed in *Petunia an2* mutants (Spelt *et al.*, 2000) and that in *Arabidopsis TT2* also has been shown to promote *TT8* expression (Baudry *et al.*, 2006).

**Fig. 7.**
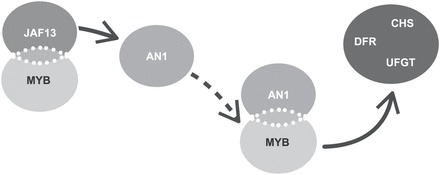
Schematic diagram representing the roles of AN1 and JAF13 in regulating anthocyanin biosynthesis and their relationships with MYB activators. Solid arrows represent direct transcriptional activation, whereas the dotted arrow shows an increased concentration of AN1 leading to the next step of transcriptional regulation. UFGT, UDP-flavonoid glycosyltransferase.

Our promoter activation analysis supports a hierarchical model where NtJAF13 is involved in the transcriptional activation of *NtAN1,* which in turn regulates the anthocyanin biosynthetic genes (Fig. 7). In tobacco, silencing of *NtAN1* by RNAi, or over-expressing *NtAN1* did not affect *NtAN1* promoter activation, suggesting that NtAN1 is not involved in activating its own promoter, unlike in *Arabidopsis*, where TT8 in combination with PAP1 or TT2 is able to regulate its own expression (Baudry *et al.*, 2006; Xu *et al.*, 2013). In contrast, the *NtJAF13* RNAi had a negative effect on *NtAN1* promoter activation (Fig. 5A) and over-expression of *NtJAF13* improved *NtAN1* promoter activation (Fig. 5B). The role of NtJAF13 as a regulator of *NtAN1* expression (in combination with AcMYB110) was further confirmed by yeast one-hybrid assay, in which AcMYB110 was able to bind the *NtAN1* promoter only in the presence of the NtJAF13 (Fig. 5D). These results, in yeast and *in planta,* establish that NtJAF13 is required by the MYB to activate *NtAN1* expression, whereas NtAN1 is not involved in its own regulation.

Given that AcMYB110 is able to activate all the promoters tested, our results suggest that the specificity of promoter recognition by the MBW complex is determined by the bHLH partner. The exact role of the bHLH in the MBW complex in different plant species is not yet clear. Studies in maize have suggested roles in reducing the inhibitory effect of single-repeat MYBs and/or in assisting transcription through aiding in chromatin accessibility (Feller *et al.*, 2011; Kong *et al.*, 2012). However, there is less known on the involvement of the bHLH in promoter *cis*-element recognition, some of which may be explained by the recent identification of further interacting proteins that alter DNA recognition by the bHLH (Kong *et al.*, 2012). Our analysis of the promoter sequences from *Arabidopsis* and four different Solanaceous species found specific target bHLH *cis*-elements: a CACGTG bHLH-binding motif was present in the promoters of the two biosynthetic genes and surrounded by several potential MYB binding sites, whereas the CAGATG and/or CATCTG motifs were specific to the *AN1* promoter (Fig. 6), supporting a model where the AN1–MYB and JAF13–MYB complexes would specifically recognise these motifs in the biosynthetic gene and the *AN1* promoters, respectively. In *Arabidopsis* the CACGTG motifs was also found in the biosynthetic gene promoters; consistent with the fact that *TT8* is able to regulate its own promoter (Baudry *et al.*, 2006), neither of the two ‘AN1-like’-specific bHLH motifs were present in the *TT8* promoter, suggesting that the model of hierarchical regulation is not well conserved outside the Solanaceae family. Future work to confirm this observation is required.

### AN1 and JAF13 regulate anthocyanin biosynthesis through a hierarchical mechanism

In the Solanaceae, specificity of the anthocyanin-activating MYBs is defined by the interaction with the bHLH partners JAF13 and AN1. The MYB, to activate anthocyanin biosynthesis, needs to associate specifically to AN1 to induce transcription of the biosynthetic genes, whereas the MYB–JAF13 complex is not able to directly activate the genes of the pathway. The MYBs therefore rely on elevating *AN1* expression so that the MYB–AN1–WD40 complex can then activate the anthocyanin response. The role of JAF13 is to enhance *AN1* expression, boosting anthocyanin biosynthesis; this is achieved by forming a complex with the MYB and activating the *AN1* promoter. JAF13 is suggested to act further upstream in the regulatory cascade in the control of anthocyanin biosynthesis.

## Supplementary data

Supplementary data are available at *JXB* online.

Figure S1. Analysis of MYB110, AN1 and JAF13 protein–protein interactions by yeast two-hybrid.

Figure S2. Colour development in flowers of stable RNAi lines targeting endogenous *NtAN1* and *NtJAF13*.

Figure S3. (A) Promoter sequence alignment of *AN1 (A), CHS (B) and DFR (C)* genes from tobacco, *Petunia*, tomato and potato.

Table S1. Oligonucleotide primer sequences.

Supplementary Data
