# Supplementary material for: In the Solanaceae, a hierarchy of bHLHs confer distinct target specificity to the anthocyanin regulatory complex
Source: J Exp Bot. 2015 Jan 26;66(5):1427–36. doi: 10.1093/jxb/eru494 (PMC4339601; doi:10.1093/jxb/eru494)
Supplement: Supplementary Data [file supp_66_5_1427__index.html]

In the Solanaceae, a hierarchy of bHLHs confer distinct target specificity to the anthocyanin regulatory complex — Supplementary Data 

# In the Solanaceae, a hierarchy of bHLHs confer distinct target specificity to the anthocyanin regulatory complex

## Supplementary Data

Data files

**Files in this Data Supplement:**

- Supplementary Data - Supplementary Data
