# Supplementary material for: In the Solanaceae, a hierarchy of bHLHs confer distinct target specificity to the anthocyanin regulatory complex
Source: J Exp Bot. 2015 Jan 26;66(5):1427–36. doi: 10.1093/jxb/eru494 (PMC4339601; doi:10.1093/jxb/eru494)
Supplement: Supplementary Data [file supp_eru494_Supplementary_Data.docx]

**Supplementary Data**

**AN1 and JAF13, two related bHLHs, confer distinct target specificity to the anthocyanin regulatory complex**

Mirco Montefiori, Cyril Brendolise, Andrew P. Dare, Kui Lin-Wang, Kevin M. Davies, Roger P. Hellens, Andrew C. Allan

**Supplementary Figure 1.** Analysis of MYB110, AN1 and JAF13 protein-protein interactions by Yeast-two-Hybrid. Various pairwise combinations (as indicated) were tested for their ability to activate the HIS3 reporter in the presence of increasing amount of 3AT. Results obtained on media containing 0, 10, 25, 50, 75 and 100 mM 3AT are presented. L, Leucine, T, tryptophan, H, histidine.

**
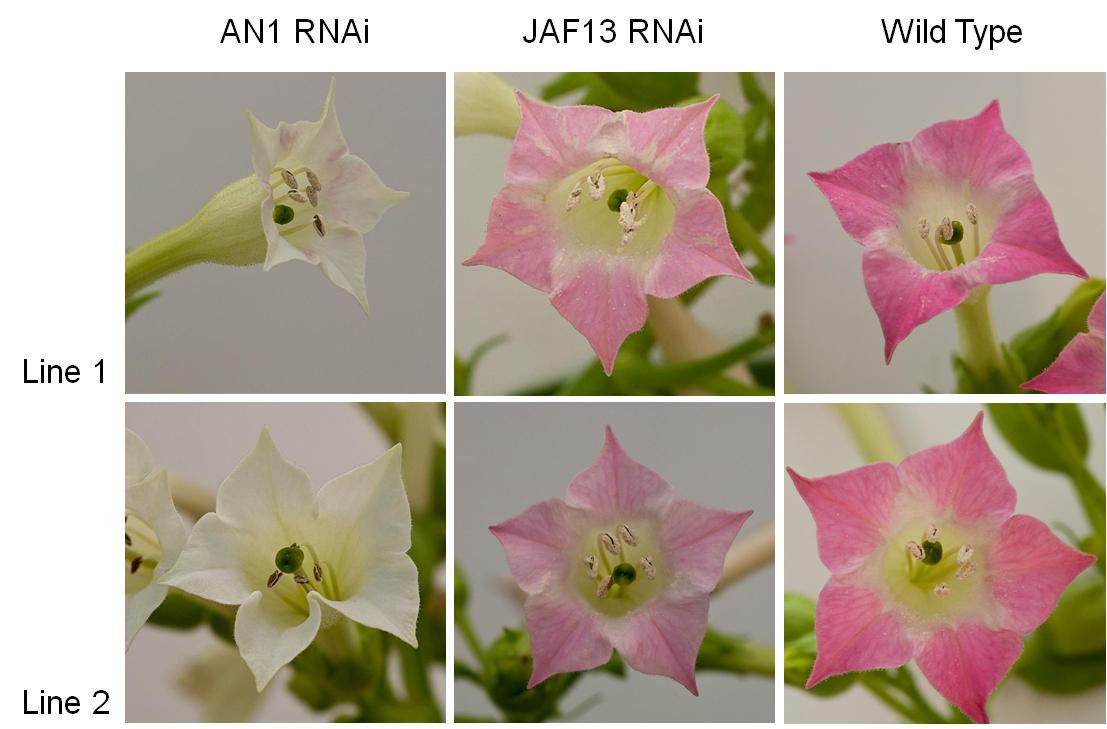
**

**Supplementary Figure 2.** Colour development in flowers of stable RNAi lines targeting endogenous *Nt AN1* and *Nt JAF13*

A

B

C

**Supplementary Figure 3.** (A) Promoter sequence alignment of *AN1 (A), CHS (B) and DFR (C)* genes from tobacco, petunia, tomato and potato. The dash-lined box identifies the 27bp conserved region in the *AN1* promoters containing the bHLH binding motif CATCTG (*) and two putative MYB binding motifs (▼) (B,C). Plain black-lined box identifies the conserved CACGTG(*) in the CHS and DFR promoters

| **Gene target** | **Forward primer (5’ – 3’)** | **Reverse primer (5’ – 3’)** |
| --- | --- | --- |
| *AcMYB110* (qPCR) | atatctacaagagaagagccgatacccaa | actgtcattgcagatagcattcaacca |
| *AcMYB110* (CDS) | caccatggaaactgttcctttaggagtgagaaag | tctcttaggtgatgattaagatg |
| *NtCHS* (qPCR) | cgggtgtgactaccaactcactaagct | gagtccttgttgtttgttcagagatcacc |
| *NtCHS* (promoter) | gtatctccaaaagtactctaaaatgtattgtatg | tccaccattttttccggcgaaaatgg |
| *NtDFR* (qPCR) | ggaactctcgatgcccaagaacac | actttgattttattagcatcataccgcc |
| *NtDFR* (promoter) | attatgtggcagacatggcagg | cccactcttctacctttcatttctgaaaatgg |
| *NtAN1* (qPCR) | ggctgctcaaaagcatactcttcactg | cgtcgtagagaaaagctaaatgagcgt |
| *NtAN1* (RNAi) | acaaagcctccattttgggtgaca | agagctgattgaatggctacaacttccac |
| *NtAN1* (promoter) | gtggtgactgcgatcaagtttca | gtctatggtgtacatcctttgcatatacaatga |
| *NtJAF13* (qPCR) | ccaggaagacagttcaggctgca | cctacaaccacaggccaaaaggc |
| *NtJAF13* (RNAi) | gatatcaagactaggaagacagttcaggtg | taccactccgtatcagtgagatcttcaggaga |

**Supplementary Table 1.** Oligonucleotide primer sequences
